# Auranofin Combination Therapy: A New Frontier in Cancer Treatment

**DOI:** 10.3390/molecules31030571

**Published:** 2026-02-06

**Authors:** Diana Laura Guzman-Gomez, Srinivasa Reddy Telukutla, Ruchika Ojha, Suresh K. Bhargava, Magdalena Plebanski

**Affiliations:** 1 Accelerator for Translational Research in Clinical Trials (ATRACT) Centre, School of Health and Biomedical Sciences, STEM College, RMIT University, Melbourne, VIC 3083, Australia; s4057062@student.rmit.edu.au; 2 Centre for Advanced Materials and Industrial Chemistry, School of Science, STEM College, RMIT University, Melbourne, VIC 3000, Australia; ruchika.ojha@rmit.edu.au (R.O.); suresh.bharagava@rmit.edu.au (S.K.B.)

**Keywords:** auranofin, gold therapy, cancer treatment, combination therapy, thioredoxin reductase, oxidative stress

## Abstract

Auranofin, a gold(I)-based compound initially developed for the treatment of rheumatoid arthritis, has emerged as a promising anticancer agent with a multimodal mechanism of action. This review comprehensively examines the therapeutic potential of auranofin in oncology focusing on its ability to synergize with conventional and emerging cancer treatments. Here, we discuss the unique pharmacological properties of auranofin, including thioredoxin reductase inhibition, reactive oxygen species induction, and modulation of key apoptotic pathways. Moreover, this article highlights new recent evidence on its ability to synergize with other cancer treatments such as chemotherapy, immunotherapy, and targeted therapies. Particular emphasis is placed on the role of auranofin in overcoming drug resistance and its potential as an adjuvant in precision medicine. By analyzing both preclinical and clinical data, this review provides critical insights into the repositioning of auranofin as a versatile component in contemporary cancer treatment paradigms, while addressing current challenges and future directions for gold-based therapeutics in oncology.

## 1. Introduction

Cancer is a group of diseases characterized by abnormal cells growing uncontrolledly forming tumors and invading surrounding and distant tissues, the latter known as metastasis. It remains one of the leading causes of death worldwide with an estimated 20 million new cases and nearly 10 million deaths in 2022 [1]. According to global cancer statistics, the most prevalent types of cancer in 2022 are lung (2.48 million cases), breast (2.29 million cases), colorectum (1.92 million) and prostate (1.46 million cases), whereas the most lethal, with 5-year survival post-diagnosis below 50%, include lung, pancreatic, liver, stomach and ovarian cancer. However, factors such as age, sex, geographic location, and socioeconomic status change the distribution of cancer around the world with different impacts and variations across populations [1,2,3,4,5].

Cancer poses a significant global health burden due to multiple factors such as lifestyle, environment, genetics and the presence and influence of specific infectious pathogens. Moreover, the lack of appropriate cancer management and palliative care in low resource settings can obstruct the progress and successful implementation of cancer treatments. Nevertheless, the disease burden might be reduced by the avoidance of risk factors, improvement of early detection techniques, and novel therapeutic approaches [2,6,7]. Current cancer therapies include surgery, chemotherapy, radiotherapy, immunotherapy, and targeted therapies, each of which changes its effectiveness depending on the type and stage of the cancer. For instance, surgery is commonly used in the early stages, but it is ineffective in the advanced stages, and the toxicity of chemotherapy and radiotherapy on normal cells becomes a limitation because it involves remarkable side effects despite being a potent treatment. In contrast, immunotherapy and targeted therapies are limited to specific conditions or cancer cell characteristics, thus limiting their utility [8].

One of the major obstacles in cancer is the recurrence or relapse of the disease accompanied by drug resistance which makes the therapy insufficient for full recovery slowing the impact of the treatment. In addition, tumor features such as heterogeneity and rapid adaptability remain paramount challenges to overcome. Therefore, the need for novel therapeutic approaches that can target cancer cells to minimize negative effects on normal tissues is important. Among the strategies used to improve the treatment effects is combination therapy, which has been broadly applied for decades due to the advantages provided by the administration of multiple therapeutic agents; for instance, the amelioration of efficacy by targeting different pathways simultaneously, the reduction in side effects by decreasing drug doses, and the attenuation of relapse and drug resistance. Hence, complementary interactions, known as synergism, are valuable for enhancing therapeutic effects in complex diseases such as cancer and controlling its progression. Moreover, this strategy coupled with a drug repurposing approach, which consists of identifying new applications for existing therapies, has recently gained momentum to accelerate the development of novel treatments in oncology, curtailing time, costs, studies, and repercussions in the long-term survival rate. In other words, given the limitations and complexity of the disease, the potential of combining novel agents or repurposed drugs with existing therapies allows the exploration of cancer from different perspectives and finding a better approach [9,10].

## 2. Auranofin

**Figure 1:**
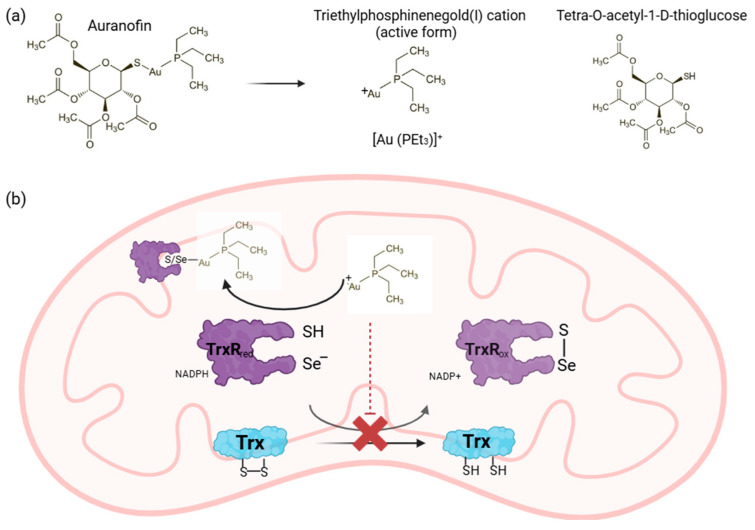
**The chemistry behind the mechanism of action of auranofin.** Chemical structure of auranofin (AF) and the generation of the active metabolite and the thioglucose (**a**); mechanistically, the active form of auranofin interacts with the selenocysteine residue in the catalytic site of TrxR impeding the reduction in Trx and consequently, ROS accumulation (**b**) [21]. Created in https://BioRender.com.

Gold complexes have gained relevance as potential anticancer drugs because of their antitumor activity against cancerous cells and unique mechanism of action which may be beneficial for established chemotherapies [11,12]. Auranofin (AF), a gold(I) compound initially developed for rheumatoid arthritis, is a prime example of a compound with anticancer potential across various cancer types including lung, ovarian, pancreatic, colon, hepatic and breast carcinoma [13,14]. The predominant molecular mechanism of AF is inhibition of thioredoxin reductase (TrxR), an enzyme overexpressed in aggressive tumors that is essential for maintaining cellular redox homeostasis [15]. Following administration, AF undergoes rapid ligand exchange reactions with biological thiols in the blood stream leading to displacement of thiosugar moiety and generation of pharmacologically active triethylphosphinegold(I) cation [Au(PEt_3_)]^+^ [16]. The thiosugar ligand contributes to pharmacokinetic properties and reduced systemic toxicity and is not directly responsible for the cytotoxicity, which is attributed primarily to the gold(I) cation. Cellular uptake of AF is thought to occur predominantly via glucose transporters, owing to its thiosugar ligand, and passive diffusion attributable to its hydrophobic character. Following the internalization, the released triethylphosphinenegold(I) cation [Au (PEt_3_)]^+^, targets redox-active selenocysteine residue located in C-terminal active site of TrxR. Nucleophilic attack of selenolate on the gold(I) center results in the formation of a stable Au-Se bond leading to irreversible inhibition of the enzyme. This disrupts electron transfer from NADPH to thioredoxin leading to functional inactivation of TrxR [16,17,18,19]. Inhibition of mitochondrial and cytosolic TRXR impairs antioxidant defense systems, leading to intracellular reactive oxygen species (ROS) accumulation, cell cycle arrest, reduced cancer cell resistance and ultimately induction of cell death (Figure 1) [13,14,20].

Notably, non-specific oxidative damage may affect healthy tissues inducing DNA damage, and excessive ROS levels can activate compensatory mechanisms that promote cell survival. Likewise, TrxR is not only involved in maintaining redox balance, but it also plays a critical role in DNA synthesis, cell proliferation and apoptosis; consequently, the inhibition of this system may contribute to unpredictable therapeutic outcomes or toxicity. Furthermore, AF’s affinity to thiol groups may cause off-target interactions with other cellular systems leading to non-specific toxicity [22].

**Figure 2:**
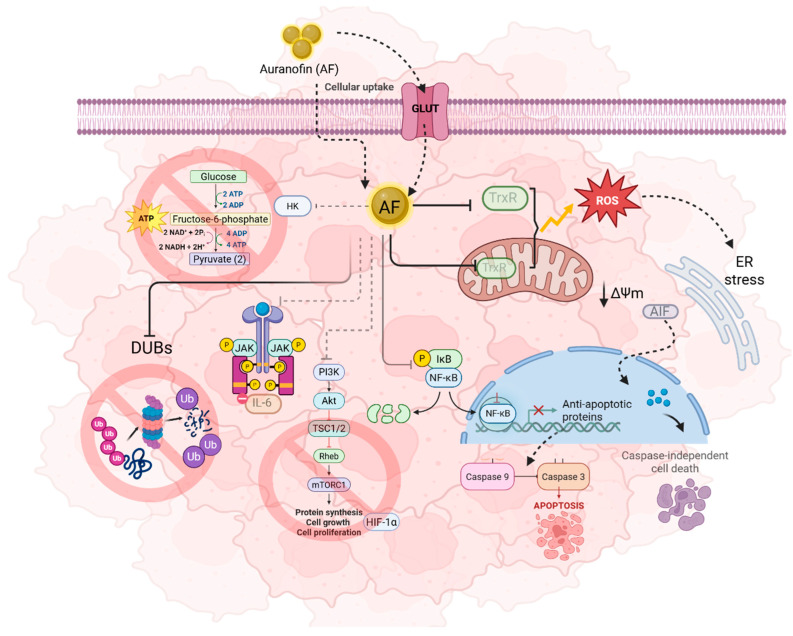
**Main biological pathways affected by Auranofin.** Cellular uptake of AF can be active by using glucose transporters (GLUTs) or passive by diffusion, internalizing the drug and metabolizing it in its active form. Subsequently, AF is involved in the disruption of a wide number of pathways inside cells, such as oxidative stress by the inhibition of TrxR enzymes which increases ROS levels and mitochondrial permeability leading to apoptosis. Additionally, AF inhibits hexokinase enzyme which participates in glycolysis, DUBs, IKK-b and NF-kb signaling pathway, activating caspase-dependent cell death. Lastly, AF interferes with JAK1-STAT3 pathway leading to anti-inflammatory and anti-proliferative properties. Created in https://BioRender.com.

Alternative signaling pathways associated with AF’s cytotoxic role in cancer as a consequence of the disruption of redox homeostasis through irreversible inhibition of TrxR include the activation of caspase-3 and -9 enzymes that triggers apoptosis, nuclear factor-kappa B (NF-kB) signaling pathway inhibition, which is crucial in inflammation and immunity, and the prevention of the degradation of the Forkhead box O3 (FOXO3) tumor suppressor factor, which in turn can modulate cytokine production, including interleukin 6 and 1β (IL-6 and IL-1β) and tumor necrosis factor alpha (TNF-α), thereby promoting cancer cell survival. Other altered mechanisms include glycolysis by hexokinase enzyme inhibition which participates in autophagy regulation, ubiquitin-proteasome system inhibition involved in cell cycle and DNA damage regulation, and downregulation of the PI3K/AKT/mTOR pathway which produce an anti-angiogenic effect [14,20,23,24] (Figure 2).

**Figure 3:**
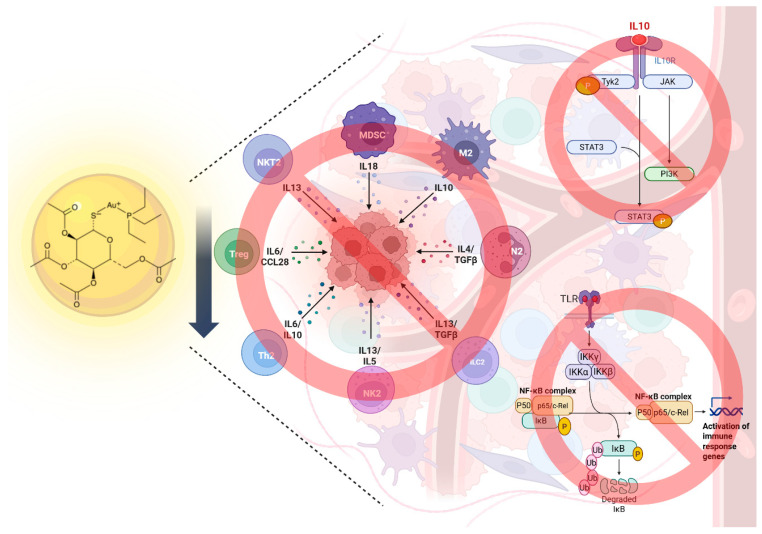
**Immunomodulatory properties of auranofin.** Immunomodulatory effects stem from auranofin’s ability to modulate immune cell function, and inflammatory signaling pathways. The primary mechanism involves NF-kB and STAT-3 pathways inhibition, resulting in the downregulation of pro-inflammatory cytokines, M2 to M1 TAMs reprogramming, and reduction in MDSCs proliferation and function, leading to restoring T cell activity. Created in https://BioRender.com.

Immunomodulation is a key focus for the development of effective cancer treatments. A reduction in immune infiltration in the tumor microenvironment (TME) has been associated with tumorigenesis, angiogenesis, metastasis and oncogenic mutations. Recent studies have suggested that AF can modulate the TME by regulating TGF-β cytokine expression, which promotes immunosuppression, while also inducing immunogenic cell death (ICD) in a dose-dependent manner [25,26]. Boullosa et al. [27] reported ICD through a significant increase in damage-associated molecular patterns (DAMPs) and mature dendritic cells (DCs) in mutant p53 lung cancer cells. Moreover, they demonstrated that some of the observed caspase-independent cell death is mediated by NK cells. In contrast, the inhibition of IkB kinase by AF modulates the activity of NF-kB, a key regulator of inflammation, tumor aggressiveness, immunosuppression and cell survival, with high levels associated with pro-inflammatory cytokines and chemokines such as IL-6, IL-1, TNF, CCL2, and CXCL12, as well as the presence of M2-polarized tumor-associated macrophages (TAMs), myeloid-derived suppressor cells (MDSCs), and Tregs (Figure 3) [23,28].

Importantly, cancer cells possess evasion strategies that limit AF’s long-term efficacy. For instance, targeting a single component of the PI3K/AKT/mTOR pathway (deregulated in cancer) can result in compensatory signaling that attenuates therapeutic efficacy [29]. Similarly, NF-κB signaling can either promote or suppress tumorigenesis depending on the microenvironment. Acute NF-κB activation during inflammation facilitates rapid immune cell recruitment and is often associated with favorable outcomes, whereas chronic activation promotes cancer cell proliferation, survival, and malignancy [30]. Therefore, understanding the complexity of these alternative pathways is critical for optimizing AF-based therapeutic strategies and minimizing off-target toxicities.

## 3. Potential of Auranofin Combination Therapy in Cancer Treatment

AF represents a potentially valuable adjunct to existing cancer treatment paradigms, particularly in overcoming resistance to other drugs, which is a significant obstacle in the current oncological treatments. Its ability to target multiple molecular pathways offers an opportunity to enhance the efficacy of established cancer therapies and open multimodal cancer treatment regimens for synergistic integration with chemotherapy, immunotherapy and targeted therapies [14]. *In vitro* and in vivo studies have demonstrated that auranofin potentiates the efficacy of chemotherapeutics, immunomodulators and targeted compounds by sensitizing current complexes and overcoming the mechanisms of drug resistance that limit the success of monotherapies [26,31].

### 3.1. Preclinical In Vitro Studies Auranofin Combination Therapy

Overall, preclinical studies have demonstrated that AF may be a valuable tool for cancer therapy by synergizing with other conventional drugs, leading to reduced tumor growth and enhanced efficacy (Table 1) [23]. Furthermore, the integration of AF can potentially reduce the need for high doses of single-agent treatments, boosting therapeutic outcomes and reducing off-target effects in multiple malignancies [31]. The following table summarizes some of the current in vitro combination studies on different cancer cell lines and their impact when combining auranofin with other drugs.

#### Potential Mechanisms of Synergistic Effects

As shown above, multiple *in vitro* studies have demonstrated significant synergy of auranofin when combined with various anticancer agents by exacerbating oxidative and proteotoxic stress, disrupting redox balance, and modulating tumor and immune signaling pathways in various types of cancer. One of them is L-BSO, a glutathione (GSH) inhibitor, which is highly elevated in cancer, is associated with tumor progression and drug resistance, and shows synergistic cytotoxicity by interfering with both TrxR and GSH antioxidant systems in ovarian, lung, pancreatic carcinoma, mesothelioma, and glioblastoma. Moreover, this combination exhibited lethality in both conventional drug-sensitive cancer and resistant cancer cells, decreasing viability by more than 20% above the other drug alone [32,34]. Potential mechanism of synergy includes the reduction in ROS scavenging capacity by blocking both antioxidant systems; additionally, it has been found that high levels of glutathione transferases are involved in metal detoxifications, which means that the inhibition of GSH may decrease gold efflux from cancer cells, leading to cytotoxic effects [52].

Menin is an essential oncogenic cofactor for the resulting mixed-lineage-leukemia (MLL) fusion protein in acute leukemia. Menin-MLL inhibitors have demonstrated effects against diverse types of cancer, including breast, ovarian and pancreatic cancer. According to Kato et al. [35], MI-463 and auranofin synergistically potentiate growth inhibition activities reducing viability by 80–90% in human ovarian, pancreatic, lung and breast cancer cells by inducing ferroptosis.

In HGSOC, approximately 50% of patients exhibit MYC amplification, an oncogenic transcription factor that plays a critical role in tumorigenesis and metabolism. Raninga et al. [36] found that auranofin is involved in glycolysis inhibition, resulting in a switch to glutamine metabolism for survival. To enhance the anti-neoplastic activity, they proposed the addition of a glutaminase inhibitor (CB-839), exerting a synergistic growth suppressive effect against MYC-high HGSOC cells by altering glutamine metabolism and glycolysis and oxidizing various proteins involved in proteasome, RNA transport, and the REDOX system.

Celecoxib, a nonsteroidal anti-inflammatory drug (NSAID) and sulfasalazine, a disease-modifying anti-rheumatic drug (DMARD) used to treat arthritis, have been shown to have anticancer activity due to their anti-inflammatory properties. Therefore, Han et al. and Li et al. [37,38] studied their synergistic effect in combination with AF demonstrating more than a 40% increase in cytotoxicity in colon and lung cancer cells by oxidative damage and ferroptosis respectively. Mechanistically, this combination induces severe oxidative stress, causing a disruption of glycolysis leading to ATP imbalance in cancer cells.

Nitazoxanide (NTZ), an antiprotozoal agent, has exerted anti-neoplastic activity in prostate, ovarian, colon, cervical, and lung cancer using a concentration between 25 and 45 μM by targeting 20S proteasome causing RE stress. Recent studies have demonstrated the advantage of AF and NTZ used together, to synergistically enhance their anti-proliferative and anti-migratory activities in anaplastic thyroid cancer, by inducing oxidative stress, cell cycle arrest in the phase G0/G1, autophagy and ferroptosis [39,53].

Mesupron, a second-generation serine protease inhibitor prodrug, has been recognized as the first class urokinase-type plasminogen activator inhibitor that is highly expressed in human breast cancer by regulating cell proliferation, invasion, and death. Therefore, Lee et al. [41] assessed the potential of Mesupron combined with AF to increase anticancer effects reporting about 60 to 70% of cell growth inhibition compared to the treatment with Mesupron alone, which showed only about 10% inhibition at 24 h. These studies further indicated a synergistic effect in one out of two breast cancer cell lines, by inducing PARP cleavage, mitochondrial disruption, and AKT phosphorylation inhibition. The accumulation of AF in the mitochondria combined with mitochondrial dysfunction caused by Mesupron triggers a synergistic effect on mitochondrial membrane potential depolarization, producing apoptosis mediated by caspase 3 activation [14].

ICG-001, an inhibitor of β-catenin/TCF mediated transcription, exhibits antitumor activity against a wide variety of cancer types including colon, pancreatic gastric and uveal carcinomas triggering cell cycle arrest and apoptosis. Lin et al. [40] tested the effect of AF and ICG-001 to evaluate their interactions and found that this combination produced better migration, colony formation and invasion suppression in colon cancer cell lines than treatment alone, inhibiting cell growth with moderate synergy (CI between 0.7 and 0.85). Moreover, it showed enhanced proliferation inhibition in 3D cell culture models by measuring the diameter changes in spheroids. In addition, they proposed that the mechanism of action might involve downregulation of p-STAT 3 and Bcl-xL and upregulation of cleaved-caspase 3, inducing apoptosis.

Spermidine, a polyamine, has been identified as a potential anticancer molecule due to its biological role in proliferation, angiogenesis, and inflammation suppressing hepatocellular carcinoma (HCC). Therefore, Hwangbo et al. [42] investigated the role of auranofin in combination with spermidine in HCC and showed more efficient toxicity against these cancer cells than the use of each drug alone in a synergic manner. Likewise, the proposed mechanism of action involves apoptosis via ROS accumulation and suppression of the PI3K/Akt signaling pathway.

Numerous preclinical studies have demonstrated that auranofin can synergize with conventional chemotherapies such as paclitaxel, doxorubicin, and cisplatin sensitizing their effects mainly attributed to AF’s ability to amplify oxidative stress. For instance, Wang et al. [51] reported 50% improvement in cytotoxicity, ROS accumulation, and cell apoptosis in liver cancer and melanoma when doxorubicin was incorporated with AF in both sensitive and resistant cell lines; Deepika et al. [50] demonstrated a synergic interaction between AF and paclitaxel in breast cancer cell lines promoting an increase in FOXO3 expression implicated in proliferation and apoptosis. Moreover, Natarajan et al. [49] illustrated the chemo-sensitizing properties of this combination to promote oxidative stress in breast cancer cells. Combining AF with cisplatin also enhances antineoplastic activity in urothelial, lung and ovarian carcinomas, by amplifying oxidative stress and mitochondrial disruption, making cells more vulnerable to cisplatin induced DNA damage [46,47,48].

The therapeutic landscape of oncology has evolved dramatically in recent years, moving beyond traditional cytotoxic agents to more targeted and immune-based treatments. Consequently, both strategies have been studied in combination with AF to disrupt the compensatory signaling pathways exerted by cancer cells. Boullosa et al. [45] highlighted the promising combination of AF and Olaparib, a PARP inhibitor, that plays a major role in DNA repair and genomic stability, demonstrating the improvement of treatment effectiveness via ROS-dependent apoptosis and ferroptosis in pancreatic and lung cancer cells. Additionally, kinase inhibitors such as Trametinib and Ibrutinib have explored a new window in cancer therapy, given that when combined with AF these drugs work by blocking enzyme decreasing growth signals, enhancing p38 MAPK signaling, inhibiting the MEK/ERK pathway, and inducing AIF-mediated apoptosis in a synergistic manner [43,44].

### 3.2. Preclinical In Vivo Studies Auranofin Combination Therapy

Similarly, combination strategies have been increasingly explored in vivo in animal models (Table 2). The following table summarizes some of the latest in vivo combination studies.

As described above, several animal model studies have confirmed the enhanced efficacy of dual treatment regimens previously shown *in vitro*, by inhibiting tumor growth without increasing systemic toxicity in models of lung, colon, and breast cancer. For instance, AF combined with vitamin C, Olaparib, celecoxib, ICG-001, sulfasalazine and nitazoxanide not only exhibited potent effect *in vitro* (Table 1), but also delayed tumor growth and metastasis in mice; these are the most promising combinations for clinical translation and confirm the mechanistic basis of synergy described by the authors including TrxR and GSH synthesis inhibition, disruption of redox homeostasis, mitochondrial dysfunction, DNA damage, cell cycle arrest, DUB system inhibition and enhanced apoptosis. Additionally, conventional chemotherapeutic drugs including cisplatin, doxorubicin and fluorouracil have shown good synergy when combined with auranofin in animal models enhancing the sensitivity and reduction in tumor growth (Table 2).

In recent years, immune check points as another therapeutic approach have been gaining importance, since upregulation of programmed death-1 receptor and ligand (PD-1/PD-L1) has been found to be a drug resistance mechanism in many types of cancer including ovarian, breast, pancreatic, colon and gastric carcinomas due to the neutralization of T cell activity [63]. Raninga et al. [63] proposed the simultaneous application of AF and an anti-PD-L1 antibody as a novel therapeutic strategy for triple-negative breast cancer sensitizing breast tumors to AF. Increased T-cell infiltration mediated by AF combined with the blockade of T-cell inactivation using PD-L1 antibodies enhances anticancer effects and reduces tumor chemoresistance, making it a promising therapeutic approach [14].

Targeted therapies such as ibrutinib, an irreversible kinase inhibitor have revealed anti-carcinogenic activity in lymphoma and leukemia by EGFR inhibition and boosting the immune response. Therefore, Hu et al. [43] tested the repercussions when combined with AF in lung xenograft models showing that this amalgamation led to 82% tumor inhibition compared to monotherapies which reduced tumor growth by approximately 60%. Likewise, VE822, a selective ATR (Ataxia-telangiectasia and Rad3-related) inhibitor, enabled complete regression of tumor xenografts when added simultaneously with auranofin, showing no significant body weight change in comparison with the control [60].

Collectively, these preclinical studies underscore the therapeutic potential of auranofin as an adjuvant therapy in combination with diverse drugs, across multiple cancer types. By targeting redox homeostasis and cellular stress responses, auranofin acts as a sensitizer to enhance the efficacy of a broad range of anticancer agents. These findings provide a strong rationale for clinical translation and further mechanistic studies to optimize combination protocols. However, the limitations of the studies summarized above should be considered. The therapeutic efficacy observed in some *in vitro* studies did not correlate with the results of corresponding in vivo studies. On the other hand, the absence of reported adverse effects is likely because most studies evaluated only body weight, without assessing other common toxicities. Additionally, the short duration of these studies may have contributed to the lack of observable physical symptoms. Therefore, long-term treatments are needed to properly assess the safety of the novel combinations. Moreover, a deeper understanding of the mechanisms of action of combination therapies is critical to ensure beneficial outcomes in clinical trials.

### 3.3. Clinical Trials

AF has been extensively supported by preclinical studies as a promising anticancerogenic drug alone or in combination with cytotoxic agents used for cancer or other types of diseases due to its established safety profile in RA and its novel supplementary mechanism of action in cancer therapy. Its translation into clinical oncology is still in phase 1 and II and ongoing trials; it is being investigated in leukemia, glioblastoma, ovarian and lung carcinoma [65]. Crucial insights into toxicity profiles, dosage optimization, progression-free survival, and safety will soon become available. Currently, four clinical trials using AF are being studied, among which two used AF as monotherapy in patients with chronic lymphocytic leukemia (CLL) (NCT01419691) and recurrent epithelial ovarian cancer (NCT01747798) without posted results. The lack of significant results may be related to the limited efficacy of AF alone at these tested doses or to the presence of mild to moderate adverse events; however, study completion suggests that the treatment was generally well tolerated and may be suitable for use as maintenance therapy [66]. Notable phase I and phase 2 trials of our interest are the combination of AF with a mTOR inhibitor, sirolimus, used in patients with ovarian cancer and subtypes of lung cancer (NCT03456700; NCT01737502), demonstrating tolerable side effects and preliminary signs of efficacy. In addition, a new protocol (CUSP9v3) has been reported to be safe against glioblastoma using a combination of nine repurposed non-oncological drugs with temozolomide including auranofin (NCT02770378). Despite early indications of tolerability and synergistic potential of AF alone or in combination, the clinical evidence remains insufficient to fully establish the therapeutic value of auranofin and guide its integration into standard cancer care [65,66,67].

Clinical data on AF are scarce, as was acknowledged in this study. Therefore, it is essential to prioritize well-designed trials that investigate other promising combinations. Given its potential to modulate the tumor microenvironment and enhance immune responses across various cancer types AF’s combination with immune checkpoint inhibitors (e.g., PD-1/PD-L1 inhibitors), chemotherapeutic agents (e.g., paclitaxel, and cisplatin) and targeted therapies (e.g., ibrutinib) require further investigation [14,23,25]. Additionally, improving targeted delivery and identifying biomarkers that can predict patient response to AF-based therapies may be explored in order to increase efficacy while reducing adverse effects [66,68].

## 4. Challenges for Combination Therapy

Combination therapy has become a cornerstone of cancer treatment, improving efficacy, reducing drug resistance, and offering synergistic benefits. From a mechanistic perspective, AF represents a promising candidate for combination therapy because its primary action on redox homeostasis and TrxR inhibition which may sensitize cancer cells to current chemotherapy. Furthermore, by disrupting other altered cellular pathways such as the ubiquitin-proteasome system, NF-kB and PI3K/AKT/mTOR pathways, AF can enhance antiproliferation and overcome resistance mechanisms. Additionally, its immunomodulatory properties may promote ICD providing a rationale for its synergy with immunotherapy [14,23].

Nevertheless, multiple challenges must be overcome for successful implementation, for instance co-administered drugs may compete for metabolic pathways or alter bioavailability, potentially leading to additive or synergistic toxicities. As a lipophilic compound, AF faces challenges in absorption, distribution, and systemic delivery, which may affect the effective concentration delivered to cancer cells in tumors, and interfere with the metabolism and clearance of other co-administered drugs. Combinations may exceed tolerability due to cumulative toxicity or off-target effects. Additionally, genetic, epigenetic and environmental variability among patients and cancer types may introduce unpredictable pharmacological behavior hindering effective dose optimization and therapeutic efficacy potentially resulting in inconsistent clinical outcomes [9,14].

For instance, clinical data (NCT01737502, NCT03456700) reported significant adverse events including gastrointestinal disorders (80%) such as diarrhea, nausea and vomiting, blood system disorders (75%) like anemia, and other general disorders including fatigue (65%), arthralgia (45%), and metabolic imbalances (15–20%) [65,66]. These toxicities underscore the necessity for careful evaluation and monitoring when combining AF with other drugs, as co-administration may exacerbate the severity of side effects.

The mitigation of these issues is critical to contributing positively to overall patient survival using novel approaches. One strategy is to improve targeting through multimodal delivery approaches, including nanoformulations such as liposomes, nanoparticles and dendrimers, which can overcome the conventional limitations by enhancing solubility, increasing drug concentration at target sites, prolonging drug half-life, and reducing systemic toxicity and multi-drug resistance [69,70].

Although combination therapy significantly enhances cancer treatment efficacy and addresses drug resistance, inherent challenges represent a substantial barrier to success. Therefore, overcoming these obstacles requires innovative strategies and further research to optimize safety and accessibility, maximize therapeutic benefits and minimize adverse effects.

## 5. Conclusions and Future Perspectives

In conclusion, innovative strategies in oncology, including the implementation of novel combination therapies, are essential to overcome the challenges in oncology. Among the emerging agents, AF, a promising antineoplastic agent, has exhibited potent sensitizing effects when combined with chemotherapeutics, targeted inhibitors, immunotherapies, and metabolic modulators in multiple preclinical models. Its mechanisms of action, including redox disruption, metabolic interference, modulation of oncogenic pathways and immunomodulation, provide a strong rationale for combination approaches that simultaneously affect the survival, proliferation, and immune evasion of cancer cells. Consequently, future research should focus on identifying optimal combination strategies, including rational pairing with agents that target complementary pathways in cancer cells. Furthermore, the development of co-delivery platforms may enhance drug accumulation, improve pharmacokinetics and mitigate drug toxicity. Additionally, personalization of treatment regimens and integration of immunomodulatory properties may expand the therapeutic window to maximize efficacy while minimizing side effects. Overall, by addressing these challenges and optimizing combination strategies, the unique mechanistic profile of AF in combination therapy has the potential to improve clinical outcomes, particularly in resistant, recurrent and advanced tumors.

## Figures and Tables

**Table 1:** *In vitro* studies of auranofin combinations in cancer.

Combination	Cancer Type	Cell Type	Key Findings	Reference
L-buthionine-sulfoximine (L-BSO) + AF	High-grade serous ovarian carcinoma (HGSOC)	PEO1, PEO4	Synergic interactions, improved cytotoxicity Elevation of ROS and TrxR inhibition	[32]
L-BSO/PPL + AF	Glioblastoma	U87MG, T98G TP53wt	The synergistic combination of AF with PPL revealed GSTP1 as a promising therapeutic vulnerability in GSCs	[33]
L-BSO + AF	Glioblastoma	EGFRwt, EGFRvIII GBM cell lines	Co-targeting TrxR and GSH systems improve the dismal survival rate in EGFR-driven cancer.	[34]
Menin-MLL inhibitors + AF	Ovarian, breast, lung and pancreatic	OVCAR-8, -3 -4; A549, LU99, LU65, PC-7; BT-549, MDA-MB-468; CFPAC-1 PANC-1, BxPC-3	Potent synergic interaction by inducing ferroptosis	[35]
Glutaminase inhibitor + AF	HGSOC	OVCAR-8, -4, -3, OAW42, PEO1, PEO4, SKOV-3, JAM	Combined therapy of GLS1 inhibitor and TrxR1 inhibitor could effectively treat MYC-high HGSOC patients	[36]
Celecoxib + AF	Colon	DLD-1, HCT116, HT-29, LOVO	Severe oxidative stress by ROS-mediated inhibition of hexokinase (HK) and disturbance of mitochondrial redox homeostasis	[37]
Sulfasalazine (SAS) + AF	Lung	Lewis lung carcinoma (LLC)	Synergistic interaction based on ferroptosis	[38]
Nitazoxanide + AF	Thyroid	8505C, C643, SW1736, THJ16T	Synergistic anticancer activity by boosting G1/G0 arrest, autophagy, apoptosis and ferroptosis.	[39]
ICG-001 + AF	Colon	HCT-116, SW-480, DLD-1	Enhanced suppression of proliferation and metastasis by STAT 3 phosphorylation inhibition	[40]
Mesupron + AF	Breast	MCF-7 MDA-MB-231	High anticancer synergy by enhanced response in cytotoxicity, apoptosis, ROS production, MPP disruption.	[41]
Spermidine + AF	Hepatocellular carcinoma	Hep3B	Synergistic anticancer activity via PI3/Akt signaling pathway reduction and ROS stimulation.	[42]
Ibrutinib + AF	*EGFR*-mutant Lung	H1975, PC9, H1650, HCC827, Calu3, H460, A549	Enhanced inhibition of the expression or phosphorylation of multiple key nodes in the AKT/mTOR and MEK/ERK pathways.	[43]
Trametinib + AF	Breast	MCF-7, MDA-MB-231	Synergistic breast cancer cell death by cell cycle arrest, mitochondrial stress and apoptosis via PARP cleavage and caspase-3/7 activation.	[44]
Olaparib + AF	Lung and pancreatic ductal adenocarcinoma	Mia-PaCa-2, Panc-1, Capan-2, BxPC-3, A549, NCI-H1975, -H2228, -H596,	Promising novel combination for mutant p53 cancers using high concentrations	[45]
Cisplatin +AF	Urothelial carcinoma	HT 1376, BFTC 909	Enhanced anti-neoplastic activity in urothelial cancer due to moderate to strong synergism	[46]
Cisplatin + AF	Lung	H69, H196	Synergistic anti-proliferative effect in SCLC Increased anti-growth activity of cisplatin	[47]
Cisplatin + AF	Ovarian	TOV112D, IGROV-1/CP	AF re-sensitizes different subtypes of EOC cells to cisplatin	[48]
Paclitaxel + AF	Breast	MCF-7, MBA-MB-231	Amplified response of paclitaxel by mitochondrial and oxidative damage	[49]
Paclitaxel + AF	Breast	MCF-7, MBA-MB-231	Sensitizing of paclitaxel via FOXO3/Nrf2/Keap1 signaling pathway regulation	[50]
Doxorubicin + AF	Melanoma, hepatocarcinoma	B16F10, MCF-7, B16F10/ADR, HepG2	Application of AF as a chemosensitizer Increment of cytotoxicity and apoptotic rates.	[51]

Only *in vitro* studies published in the last decade are included in this table.

**Table 2:** *In vivo* studies of auranofin combinations in cancer.

Combination	Cancer Type	Mice Type	Key Findings	Reference
Vitamin C + AF	Triple-negative breast cancer	MDA-MB-231 xenografts in nude mice	AUF/VC combinations revealed higher therapeutic efficacy than single drugs.	[54]
Sorafenib + AF	SK-Hep1^OE^ human liver cancer xenografts and AML leukemia model	Female BALB/c nude mice aged 5–6 weeks	AF enhanced effect against sorafenib-resistant cancer in vivo by mitochondrial TXNRD3 overexpression.	[55]
Celecoxib + AF	Colon cancer	Female athymic nude mice	Delayed tumor growth in vivo by severe oxidative stress.	[37]
Sulfasalazine + AF	Lung cancer	Female C57BL/6JJcl mice	Lung cancer growth and Lewis lung carcinoma survival suppression.	[38]
BSO + AF	Head and neck cancer	Thymic BALB/c male nude mice	Significant inhibition of HNC in vivo growth.	[56]
ICG-001 + AF	Colon cancer	Male null-BALB/c mice	Significant suppression of STAT3 phosphorylation and the downstream mediator Bcl-xL.	[40]
Olaparib + AF	Lung cancer	129 mouse model	Simultaneous treatment significantly delayed tumor growth in a mutant p53 syngeneic lung adenocarcinoma mouse model.	[45]
Celecoxib + AF	Osteosarcoma, Synovial sarcoma	C3H/HeSlc mice, nu/nu mice	Significant inhibition of osteosarcoma local progression and pulmonary metastasis.	[57,58]
Nitazoxanide	Thyroid cancer	BALB/c, nu/nu mice	Synergistic anticancer activity.	[39]
Disulfiram + AF	Hepatocellular carcinoma	HepG2 and SMMC-7721 xenografts	Co-treatment inhibited tumor growth in vivo by DUBs system inhibition.	[59]
VE822 + AF	Osteosarcoma and breast cancer	Female BALB/c nude mice	Co-administration induces regressions of tumor xenografts.	[60]
5Z-7-oxozeaenol + AF	Colon cancer	Athymic, female nu/nu nude mice	Improved efficacy of 5Z-7-oxozeaenol in vivo.	[61]
Ibrutinib + AF	Lung adenocarcinoma	Female nude mice	Enhanced in vivo efficacy of ibrutinib in *EGFR*-mutant lung cancer.	[43]
Adriamycin + AF	Lung cancer	Athymic mice	Enhanced therapeutic activity reducing potential tumor growth.	[62]
Anti-PD-L1 + AF	Triple-negative breast cancer	Female immunocompromised NOD/SCID mice, female immunocompetent BALB/c mice	Significant suppression of 4T1.2 tumor growth compared to a single agent. Enhanced efficacy of PD-L1 targeting therapy by Trx system disruption.	[63]
Cisplatin + AF	Lung cancer	Athymic nude mice	Potent reduction in SCLC tumor growth in vivo.	[47]
5-fluorouracil	Colorectal cancer	Male BALB/c nude mice	AF enhances the sensitivity of SW620/5-FU cells to 5-FU treatment in vivo.	[64]

*In vivo* studies reported in this table were published in the last decade.

## Data Availability

No new data were created or analyzed in this study.

## References

[1] Bray F., Laversanne M., Sung H.Y.A., Ferlay J., Siegel R.L., Soerjomataram I., Jemal A. (2024). Global cancer statistics 2022: GLOBOCAN estimates of incidence and mortality worldwide for 36 cancers in 185 countries. CA-Cancer J. Clin..

[2] Schwartz S.M. (2024). Epidemiology of Cancer. Clin. Chem..

[3] IARC Cancer Today—Data Visualization Tool. https://gco.iarc.who.int/today/en/dataviz/pie?mode=cancer&group_populations=1&multiple_populations=1&show_table_pie=1&details_other=1.

[4] WHO Cancer. https://www.who.int/news-room/fact-sheets/detail/cancer#:~:text=Tobacco%20use%2C%20alcohol%20consumption%2C%20unhealthy,%2D%20and%20middle%2Dincome%20countries..

[5] ACS. American Cancer Society Cancer Types. https://www.cancer.org/cancer/types.html.

[6] WHO Global Cancer Burden Growing Amidst Mounting Need for Services. https://www.who.int/news/item/01-02-2024-global-cancer-burden-growing--amidst-mounting-need-for-services.

[7] Wu Z.H., Xia F.N., Lin R. (2024). Global burden of cancer and associated risk factors in 204 countries and territories, 1980–2021: A systematic analysis for the GBD 2021. J. Hematol. Oncol..

[8] Debela D.T., Muzazu S.G.Y., Heraro K.D., Ndalama M.T., Mesele B.W., Haile D.C., Kitui S.K., Manyazewal T. (2021). New approaches and procedures for cancer treatment: Current perspectives. Sage Open Med..

[9] Mokhtari R.B., Homayouni T.S., Baluch N., Morgatskaya E., Kumar S., Das B., Yeger H. (2017). Combination therapy in combating cancer. Oncotarget.

[10] Deben C., Boullosa L.F., Fortes F.R., de la Hoz E.C., Le Compte M., Seghers S., Peeters M., Vanlanduit S., Lin A.B.H., Dijkstra K.K. (2024). Auranofin repurposing for lung and pancreatic cancer: Low CA12 expression as a marker of sensitivity in patient-derived organoids, with potentiated efficacy by AKT inhibition. J. Exp. Clin. Cancer Res..

[11] Moreno-Alcantar G., Picchetti P., Casini A. (2023). Gold Complexes in Anticancer Therapy: From New Design Principles to Particle-Based Delivery Systems. Angew. Chem. Int. Ed..

[12] Falchetti M., Delgobo M., Zancanaro H., Almeida K., das Neves R.N., Dos Santos B., Stefanes N.M., Bishop A., Santos-Silva M.C., Zanotto-Filho A. (2023). Omics-based identification of an NRF2-related auranofin resistance signature in cancer: Insights into drug repurposing. Comput. Biol. Med..

[13] Onodera T., Momose I., Kawada M. (2019). Potential Anticancer Activity of Auranofin. Chem. Pharm. Bull..

[14] Abdalbari F.H., Telleria C.M. (2021). The gold complex auranofin: New perspectives for cancer therapy. Discov. Oncol..

[15] Saei A.A., Gullberg H., Sabatier P., Beusch C.M., Johansson K., Lundgren B., Arvidsson P.I., Arner E.S.J., Zubarev R.A. (2020). Comprehensive chemical proteomics for target deconvolution of the redox active drug auranofin. Redox Biol..

[16] Snyder R.M., Mirabelli C.K., Crooke S.T. (1986). Cellular association, intracellular distribution, and efflux of auranofin via sequential ligand exchange reactions. Biochem. Pharmacol..

[17] Ma J., van de Sande W., Biersack B. (2025). Exploring a Therapeutic Gold Mine: The Antifungal Potential of the Gold-Based Antirheumatic Drug Auranofin. Int. J. Mol. Sci..

[18] Shen S.Y., Shen J., Luo Z., Wang F.D., Min J.X. (2023). Molecular mechanisms and clinical implications of the gold drug auranofin. Coord. Chem. Rev..

[19] Kean W.F., Hart L., Buchanan W.W. (1997). Auranofin. Br. J. Rheumatol..

[20] Roder C., Thomson M.J. (2015). Auranofin: Repurposing an Old Drug for a Golden New Age. Drugs R&D.

[21] Gómez-Espina J., Blanco-González E., Montes-Bayón M., Sanz-Medel A. (2016). HPLC-ICP-MS for simultaneous quantification of the total and active form of the thioredoxin reductase enzyme in human serum using auranofin as an activity-based probe. J. Anal. At. Spectrom..

[22] Patial T., Sharma N., Sharma K., Mishra V. (2024). Generation and Application of Reactive Oxygen Radicals in Cancer Treatment. Chemistryselect.

[23] Gamberi T., Chiappetta G., Fiaschi T., Modesti A., Sorbi F., Magherini F. (2022). Upgrade of an old drug: Auranofin in innovative cancer therapies to overcome drug resistance and to increase drug effectiveness. Med. Res. Rev..

[24] Hsieh M.S., Ling H.H., Setiawan S.A., Hardianti M.S., Fong I.H., Yeh C.T., Chen J.H. (2024). Therapeutic targeting of thioredoxin reductase 1 causes ferroptosis while potentiating anti-PD-1 efficacy in head and neck cancer. Chem.-Biol. Interact..

[25] Singh N., Baby D., Rajguru J.P., Patil P.B., Thakkannavar S.S., Pujari V.B. (2019). Inflammation and Cancer. Ann. Afr. Med..

[26] Massai L., Cirri D., Marzo T., Messori L. (2022). Auranofin and its analogs as prospective agents for the treatment of colorectal cancer. Cancer Drug Resist..

[27] Boullosa L.F., Van Loenhout J., Flieswasser T., De Waele J., Hermans C., Lambrechts H., Cuypers B., Laukens K., Bartholomeus E., Siozopoulou V. (2021). Auranofin reveals therapeutic anticancer potential by triggering distinct molecular cell death mechanisms and innate immunity in mutant p53 non-small cell lung cancer. Redox Biol..

[28] Shi S.Y., Ou X.H., Liu C., Li R., Zheng Q.J., Hu L.M. (2025). NF-κB signaling and the tumor microenvironment in osteosarcoma: Implications for immune evasion and therapeutic resistance. Front. Immunol..

[29] Li H., Hu J., Wu S., Wang L., Cao X., Zhang X., Dai B., Cao M., Shao R., Zhang R. (2016). Auranofin-mediated inhibition of PI3K/AKT/mTOR axis and anticancer activity in non-small cell lung cancer cells. Oncotarget.

[30] Mao H., Zhao X., Sun S.C. (2025). NF-kappaB in inflammation and cancer. Cell. Mol. Immunol..

[31] Chmelyuk N., Kordyukova M., Sorokina M., Sinyavskiy S., Meshcheryakova V., Belousov V., Abakumova T. (2025). Inhibition of Thioredoxin-Reductase by Auranofin as a Pro-Oxidant Anticancer Strategy for Glioblastoma: In Vitro and In Vivo Studies. Int. J. Mol. Sci..

[32] Abdalbari F.H., Martinez-Jaramillo E., Forgie B.N., Tran E., Zorychta E., Goyeneche A.A., Sabri S., Telleria C.M. (2023). Auranofin Induces Lethality Driven by Reactive Oxygen Species in High-Grade Serous Ovarian Cancer Cells. Cancers.

[33] Jamali F., Lan K., Daniel P., Petrecca K., Sabri S., Abdulkarim B. (2024). Synergistic Dual Targeting of Thioredoxin and Glutathione Systems Irrespective of p53 in Glioblastoma Stem Cells. Antioxidants.

[34] Martinez-Jaramillo E., Jamali F., Abdalbari F.H., Abdulkarim B., Jean-Claude B.J., Telleria C.M., Sabri S. (2024). Pro-Oxidant Auranofin and Glutathione-Depleting Combination Unveils Synergistic Lethality in Glioblastoma Cells with Aberrant Epidermal Growth Factor Receptor Expression. Cancers.

[35] Kato I., Kasukabe T., Kumakura S. (2020). Menin-MLL inhibitors induce ferroptosis and enhance the anti-proliferative activity of auranofin in several types of cancer cells. Int. J. Oncol..

[36] Raninga P.V., He Y.W., Datta K.K., Lu X., Maheshwari U.R., Venkat P., Mayoh C., Gowda H., Kalimutho M., Hooper J.D. (2023). Combined thioredoxin reductase and glutaminase inhibition exerts synergistic anti-tumor activity in MYC-high high-grade serous ovarian carcinoma. Mol. Ther..

[37] Han Y., Chen P., Zhang Y.Y., Lu W.H., Ding W.W., Luo Y., Wen S.J., Xu R.H., Liu P.P., Huang P. (2019). Synergy between Auranofin and Celecoxib against Colon Cancer In Vitro and In Vivo through a Novel Redox-Mediated Mechanism. Cancers.

[38] Li H., Li S.R., Kanamori Y., Liu S.S., Moroishi T. (2025). Auranofin resensitizes ferroptosis-resistant lung cancer cells to ferroptosis inducers. Biochem. Biophys. Res. Commun..

[39] Ghosh C., Gunda V., Hu J.N., Zhang L., Zhang Y.Q., Shen M., Kebebew E. (2023). Combination nitazoxanide and auranofin treatment has synergistic anticancer activity in anaplastic thyroid cancer and act by enhanced activation of multiple cell death pathways. Cancer Res..

[40] Lin Z.Y., Li Q.Q., Zhao Y., Lin Z.X., Cheng N., Zhang D., Liu G., Lin J.H., Zhang H., Lin D.G. (2021). Combination of Auranofin and ICG-001 Suppress the Proliferation and Metastasis of Colon Cancer. Front. Oncol..

[41] Lee J.E., Kwon Y.J., Baek H.S., Ye D.J., Cho E., Choi H.K., Oh K.S., Chun Y.J. (2017). Synergistic induction of apoptosis by combination treatment with mesupron and auranofin in human breast cancer cells. Arch. Pharm. Res..

[42] Hwangbo H., Kim D.H., Kim M.Y., Ji S.Y., Bang E., Hong S.H., Choi Y.H., Cheong J.H. (2023). Auranofin accelerates spermidine-induced apoptosis via reactive oxygen species generation and suppression of PI3K/Akt signaling pathway in hepatocellular carcinoma. Fish. Aquat. Sci..

[43] Hu J., Zhang H.J., Cao M.R., Wang L., Wu S.H., Fang B.L. (2018). Auranofin Enhances Ibrutinib’s Anticancer Activity in EGFR-Mutant Lung Adenocarcinoma. Mol. Cancer Ther..

[44] Joo M.K., Shin S., Ye D.J., An H.G., Kwon T.U., Baek H.S., Kwon Y.J., Chun Y.J. (2021). Combined treatment with auranofin and trametinib induces synergistic apoptosis in breast cancer cells. J. Toxicol. Environ. Health A.

[45] Boullosa L.F., Van Loenhout J., Flieswasser T., Hermans C., Merlin C., Lau H.W., Marcq E., Verschuuren M., De Vos W.H., Lardon F. (2023). Auranofin Synergizes with the PARP Inhibitor Olaparib to Induce ROS-Mediated Cell Death in Mutant p53 Cancers. Antioxidants.

[46] Chen S.Y., Chao C.N., Huang H.Y., Fang C.Y. (2022). Auranofin induces urothelial carcinoma cell death via reactive oxygen species production and synergy with cisplatin. Oncol. Lett..

[47] Liu X.L., Wang W., Yin Y.P., Li M., Li H., Xiang H., Xu A., Mei X.D., Hong B., Lin W.C. (2019). A high-throughput drug screen identifies auranofin as a potential sensitizer of cisplatin in small cell lung cancer. Investig. New Drug.

[48] Abdalbari F.H., Forgie B.N., Zorychta E., Goyeneche A.A., Noman A.S.M., Telleria C.M. (2025). The gold complex auranofin sensitizes platinum resistant epithelial ovarian cancer cells to cisplatin. Biochem. Biophys. Rep..

[49] Natarajan D., Prasad N.R., Sudharsan M., Bharathiraja P., Lakra D.S. (2023). Auranofin sensitizes breast cancer cells to paclitaxel chemotherapy by disturbing the cellular redox system. Cell Biochem. Funct..

[50] Deepika N., Prasad N.R., Radhiga T. (2024). Auranofin sensitizes breast cancer cells to paclitaxel mediated cell death via regulating FOXO3/Nrf2/Keap1 signaling pathway. Cell Biochem. Funct..

[51] Wang X.F., Liu Y.N., Zhang W.Q., Li Z.D., Li S., Chen J.X., Li Q., Suo X.M., Zeng Y.Q., Zhang G.F. (2025). Beyond gold: The chemoenhancing mechanism and therapeutic potential of auranofin in melanoma. Cancer Biol. Med..

[52] Chiappetta G., Gamberi T., Faienza F., Limaj X., Rizza S., Messori L., Filomeni G., Modesti A., Vinh J. (2022). Redox proteome analysis of auranofin exposed ovarian cancer cells (A2780). Redox Biol..

[53] Lu Z.R., Li X.A., Li K.B., Ripani P., Shi X.M., Xu F.R., Wang M.P., Zhang L.R., Brunner T., Xu P. (2022). Nitazoxanide and related thiazolides induce cell death in cancer cells by targeting the 20S proteasome with novel binding modes. Biochem. Pharmacol..

[54] Hatem E., Azzi S., El Banna N., He T.T., Heneman-Masurel A., Vernis L., Baïlle D., Masson V., Dingli F., Loew D. (2019). Auranofin/Vitamin C: A Novel Drug Combination Targeting Triple-Negative Breast Cancer. JNCI-J. Natl. Cancer Inst..

[55] Liu X.X., Zhang Y.Y., Lu W.H., Han Y., Yang J., Jiang W.Y., You X., Luo Y., Wen S.J., Hu Y.M. (2020). Mitochondrial TXNRD3 confers drug resistance via redox-mediated mechanism and is a potential therapeutic target in vivo. Redox Biol..

[56] Roh J.L., Jang H., Kim E.H., Shin D. (2017). Targeting of the Glutathione, Thioredoxin, and Nrf2 Antioxidant Systems in Head and Neck Cancer. Antioxid. Redox Signal..

[57] Kinoshita H., Kinoshita S., Kamoda H., Hagiwara Y., Ohtori S., Yonemoto T. (2024). The Combination of Auranofin and Celecoxib Suppresses Local Synovial Sarcoma Progression Ιn Vivo. Anticancer Res..

[58] Kinoshita H., Kinoshita S., Kamoda H., Hagiwara Y., Ohtori S., Yonemoto T. (2024). Combined Auranofin and Celecoxib Suppresses the Local Progression and Pulmonary Metastases of Osteosarcoma In Vivo. Anticancer Res..

[59] Huang H.B., Liao Y.N., Liu N.N., Hua X.L., Cai J.Y., Yang C.S., Long H.D., Zhao C., Chen X., Lan X.Y. (2016). Two clinical drugs deubiquitinase inhibitor auranofin and aldehyde dehydrogenase inhibitor disulfiram trigger synergistic anti-tumor effects in vitro and in vivo. Oncotarget.

[60] Zhang S., Zhao Y., Wang X.Q., Qi C., Tian J.L., Zou Z.H. (2023). Synergistic lethality between auranofin-induced oxidative DNA damage and ATR inhibition in cancer cells. Life Sci..

[61] Hrabe J.E., O’Leary B.R., Fath M.A., Rodman S.N., Button A.M., Domann F.E., Spitz D.R., Mezhir J.J. (2015). Disruption of thioredoxin metabolism enhances the toxicity of transforming growth factor β-activated kinase 1 (TAK1) inhibition in KRAS-mutated colon cancer cells. Redox Biol..

[62] Hou G.X., Liu P.P., Zhang S.Y., Yang M.Q., Liao J.W., Yang J., Hu Y.M., Jiang W.Q., Wen S.J., Huang P. (2018). Elimination of stem-like cancer cell side-population by auranofin through modulation of ROS and glycolysis. Cell Death Dis..

[63] Raninga P.V., Lee A.C., Sinha D., Shih Y.Y., Mittal D., Makhale A., Bain A.L., Nanayakarra D., Tonissen K.F., Kalimutho M. (2020). Therapeutic cooperation between auranofin, a thioredoxin reductase inhibitor and anti-PD-L1 antibody for treatment of triple-negative breast cancer. Int. J. Cancer.

[64] Liu C., Zhao Y., Wang J.N., Yang Y., Zhang Y., Qu X.L., Peng S.S., Yao Z.Y., Zhao S.L., He B.S. (2020). FoxO3 reverses 5-fluorouracil resistance in human colorectal cancer cells by inhibiting the Nrf2/TR1 signaling pathway. Cancer Lett..

[65] NIH Clinical Trials, National Library of Medicine. https://www.clinicaltrials.gov/search?cond=cancer&term=auranofin.

[66] Giorgini D., Chiaverini L., Viviano M., Belvedere R., Salerno S., Baglini E., Da Settimo F., Marzo T., Taliani S., Barresi E. (2025). Repurposing Auranofin for Oncology and Beyond: A Brief Overview of Clinical Trials as Mono- and Combination Therapy. Pharmaceuticals.

[67] Halatsch M.E., Kast R.E., Karpel-Massler G., Mayer B., Zolk O., Schmitz B., Scheuerle A., Maier L., Bullinger L., Mayer-Steinacker R. (2021). A phase Ib/IIa trial of 9 repurposed drugs combined with temozolomide for the treatment of recurrent glioblastoma: CUSP9v3. Neuro-Oncol. Adv..

[68] Zhou L., Liu H., Liu K., Wei S. (2021). Gold Compounds and the Anticancer Immune Response. Front. Pharmacol..

[69] Nastiuk K.L., Krolewski J.J. (2016). Opportunities and challenges in combination gene cancer therapy. Adv. Drug Deliv. Rev..

[70] Obidiro O., Battogtokh G., Akala E.O. (2023). Triple Negative Breast Cancer Treatment Options and Limitations: Future Outlook. Pharmaceutics.

